# Outcomes Following Constipation Treatments Between Parkinson’s Disease and Non‐Parkinson’s Disease Patients Evaluated by the Constipation Scoring System

**DOI:** 10.1155/padi/1105437

**Published:** 2026-02-23

**Authors:** Kulthida Methawasin, Atip Krittayasingh, Kitsarawut Khuancharee, Piyanant Chonmaitree, Monton Wongwandee

**Affiliations:** ^1^ Department of Internal Medicine, HRH Princess Maha Chakri Sirindhorn Medical Center, Faculty of Medicine, Srinakharinwirot University, Ongkharak, Nakhon Nayok, Thailand, swu.ac.th; ^2^ Department of Preventive and Social Medicine, Faculty of Medicine, Srinakharinwirot University, Ongkharak, Nakhon Nayok, Thailand, swu.ac.th

**Keywords:** chronic symptoms, constipation, Constipation Scoring System, dietary habits, laxative use, Parkinson’s disease, water intake, well-being

## Abstract

**Objectives:**

The Constipation Scoring System (CSS) is a validated tool for assessing constipation severity and has been previously applied in Parkinson’s disease (PD) populations. However, comparative data on post‐treatment CSS outcomes between individuals with and without PD remain lacking. This study aimed to compare post‐treatment constipation severity between PD and non‐PD patients in real‐world clinical settings, with particular focus on neurology and gastroenterology outpatient clinics.

**Methods:**

This retrospective chart review included 67 patients with PD from a neurology clinic and 50 non‐PD patients with constipation from a gastroenterology clinic. Baseline characteristics were retrieved from electronic medical records. Follow‐up assessments were conducted through direct or telephone interviews to evaluate constipation severity using the CSS. Additional data were collected on patients’ self‐reported intake of water, coffee, carbohydrates, and fiber, as well as exercise habits.

**Results:**

Post‐treatment CSS scores did not differ significantly between groups (PD: 6.07 ± 3.57 vs. non‐PD: 5.24 ± 2.84; *p* = 0.172), with most participants classified as having mild constipation. No significant differences were observed in daily water, coffee, or fiber intake, or in exercise habits. However, non‐PD patients reported significantly higher carbohydrate intake compared to PD patients (*p* = 0.003). PD patients more frequently reported long‐standing constipation symptoms (≥ 6 years) than non‐PD patients (*p* < 0.001). Patterns of laxative use also differed: while sennosides were most commonly used in both groups, non‐PD patients more frequently used lactulose and mucilin, whereas PD patients more commonly used Unison enemas (*p* = 0.020) and milk of magnesia (*p* = 0.070).

**Conclusion:**

Although constipation severity and treatment outcomes were comparable between PD and non‐PD patients, PD patients more often experienced long‐standing symptoms and demonstrated distinct patterns of laxative use. Prospective studies are warranted to evaluate standardized treatment protocols to better clarify treatment outcomes and inform clinical practice.

## 1. Introduction

Parkinson’s disease (PD) is characterized by neurodegeneration of the substantia nigra pars compacta and presents with a combination of motor and nonmotor symptoms. Among the nonmotor features, constipation is particularly common, with a prevalence nearly three times higher in PD patients than in individuals without PD [1]. The underlying mechanisms linking PD and constipation are primarily attributed to delayed colonic transit and pelvic floor dyssynergia, particularly puborectalis dysfunction. However, the condition may be exacerbated by the concomitant use of antiparkinsonian, analgesic, or antidepressant medications.

Constipation in PD should be diagnosed using the Rome IV criteria, which incorporate subjective symptom reporting, clinical history, and physical examination [2]. Although comprehensive investigations such as colonoscopy or functional tests can elucidate the pathophysiology of constipation, they are typically reserved for cases with suspected structural abnormalities, refractory symptoms, or possible malignancy. In routine practice, the Rome IV criteria are often used to diagnose functional constipation. These criteria include the presence of at least two of the following symptoms occurring in more than 25% of defecations: (1) straining, (2) lumpy or hard stools (Bristol Stool Form Scale types 1–2), (3) sensation of incomplete evacuation, (4) sensation of anorectal obstruction, (5) need for manual maneuvers to facilitate defecation, (6) fewer than three spontaneous bowel movements per week, (7) rare loose stools without laxatives, and (8) insufficient criteria for irritable bowel syndrome. The symptoms must have been present for the previous 3 months, with onset at least 6 months before diagnosis. Other potential causes, including opioid‐induced constipation, should also be ruled out. Although the Rome IV criteria remain the diagnostic standard, their application in neurology or movement disorder clinics can be limited by time constraints and the complexity of patient care. Additionally, the subjective estimation required by these criteria may vary depending on patients’ perceptions, potentially reducing reliability in certain clinical settings.

As an alternative, the Constipation Scoring System (CSS), developed and published in 1996, offers a standardized, symptom‐based assessment of constipation severity [3]. The CSS includes eight domains: frequency of bowel movements, painful evacuation, sensation of incomplete evacuation, abdominal pain, time spent in the lavatory per attempt, need for defecation assistance, number of unsuccessful evacuation attempts per 24 h, and duration of constipation. The total score ranges from 0 to 30, with a score of 15 or higher indicative of significant constipation. The CSS has been validated and previously used in PD populations, demonstrating reliable measurement of constipation severity [4]. However, to date, no published data compare CSS scores after constipation treatment in PD versus non‐PD patients. Therefore, the primary objective of this study was to compare post‐treatment CSS scores between PD and non‐PD patients in real‐world clinical settings, specifically within neurology and gastroenterology outpatient clinics. We hypothesized that constipation in PD would be more refractory to treatment, reflected by higher post‐treatment CSS scores in PD patients compared to those in non‐PD patients. This retrospective chart review was designed to reflect real‐world outcomes without the constraints of a formal interventional protocol.

## 2. Methods

### 2.1. Study Population and Design

This retrospective chart review was conducted at the HRH Princess Maha Chakri Sirindhorn Medical Center, Srinakharinwirot University, between March 2019 and April 2020. A total of 67 PD patients and 50 non‐PD patients were selected through purposive sampling based on the inclusion and exclusion criteria defined for each group. Inclusion criteria of the PD group were (1) age 20 years old and above, (2) clinical diagnosis of PD, and (3) constipation. Patients with a history of stroke or repeated stroke, head injury, encephalitis, drug‐induced parkinsonism, corticobasal syndrome, progressive supranuclear gaze palsy, cerebellar syndrome, early autonomic dysfunction with documented orthostatic hypotension, Alzheimer’s disease, and communicating or normal pressure hydrocephalus were excluded. The inclusion criteria for the non‐PD group were (1) age 20 years and above and (2) constipation. Irritable bowel syndrome, colorectal cancer, colonic obstruction, and medication‐associated constipation patients were excluded. All PD patients were clinically diagnosed according to the UK Brain Bank criteria [5] by a certified neurologist (KM). Neuroimaging of volunteers in the PD group was also reviewed to confirm that there were no anatomical abnormalities or intracranial space‐occupying lesions. The research team retrospectively reviewed the history records from the electronic medical record system. The gastroenterologist (CP) applied Rome IV criteria to confirm the diagnosis of constipation in all volunteers. There was no CSS score recorded for volunteers’ first visits because this evaluation had never been used in the daily practice of MSMC.

### 2.2. Demographic and Clinical Measurements

Demographic and clinical data were collected for all participants, including sex, age, laxative use, use of digital or manual assistance for defecation, daily water intake, monthly coffee consumption, estimated daily carbohydrate and fiber intake, smoking status, alcohol consumption, history of other gastrointestinal conditions, physical activity, and comorbidities. Daily water intake was estimated using a 600 mL water bottle as a reference. Coffee intake was recorded as the number of cups consumed per month. Carbohydrate intake was estimated subjectively, with one ladle of steamed rice considered equivalent to 60 g of carbohydrates. Daily rice consumption was recorded in ladles and converted into grams. Fiber intake was reported as the percentage of vegetables and fruits consumed per day.

For participants in the PD group, additional clinical data were collected, including age at PD onset, disease duration, onset of constipation, Hoehn and Yahr (H&Y) stage [6], levodopa equivalent dose (LED), and Montreal Cognitive Assessment (MoCA) scores. Permission of MoCA was granted to use in this research. LED was calculated using an established online calculator [7].

Constipation treatments in this study were based on real‐world clinical practices from both the neurology and gastroenterology clinics. Management strategies included both pharmacological and nonpharmacological interventions. Pharmacological treatments involved laxative medications prescribed by physicians. A survey of available medications at HRH Princess Maha Chakri Sirindhorn Medical Center (MSMC) at the time of the study identified the following commonly used agents: sennosides, lactulose, milk of magnesia, bisacodyl suppository, mucilin, and Unison enema. Specific laxatives with established efficacy in PD, such as macrogol, prucalopride, and lubiprostone, were not available. Thai herbal medicines with laxative effects were also included where applicable.

Nonpharmacological management included routine patient education delivered by outpatient department nurses, focusing on increasing daily vegetable and fruit consumption, maintaining a water intake of at least 2 L per day, and encouraging regular exercise. Types of physical activity varied among patients and included walking, jogging, stretching, and household tasks such as gardening or cleaning. Exercise habits were assessed based on self‐reported frequency (days per week) and duration (minutes per day).

All participants received both pharmacological and nonpharmacological treatment for at least 3 months. Post‐treatment constipation severity was assessed using the CSS, administered either in person or via telephone interviews during the COVID‐19 pandemic quarantine period. Interviews were conducted by a trained neurology resident (KA) and included patients or their caregivers as appropriate.

### 2.3. Data Analysis

Continuous variables were summarized as mean ± standard deviation (SD) for normally distributed data and as median with interquartile range (IQR) for non‐normally distributed data. Categorical variables were presented as frequencies and percentages. The Shapiro–Wilk test was used to assess the normality of continuous variables. Comparisons between the PD and non‐PD groups were performed using independent *t*‐tests for normally distributed continuous variables and the Mann–Whitney *U* test for non‐normally distributed variables. Categorical variables were analyzed using the chi‐squared test or Fisher’s exact test, as appropriate. All statistical analyses were performed using SPSS version 22.0 (IBM Corp., Armonk, NY, USA). A two‐tailed *p* value of < 0.05 was considered statistically significant.

## 3. Results

### 3.1. Baseline Characteristics

A total of 117 patients with constipation were included in this retrospective study. Of these, 67 patients (57.26%) had a confirmed diagnosis of PD, while 50 patients (42.74%) served as the non‐PD comparison group. The mean age of PD patients was significantly higher than that of non‐PD patients (71.38 ± 10.88 years vs. 65.10 ± 15.64 years; *p* = 0.036). Among PD patients, the mean age at disease onset was 64.75 ± 11.61 years (median = 67; range: 36–82), and the mean duration of PD treatment was 6.63 ± 3.71 years (median = 6; range: 1–20). In terms of disease severity, most PD patients were classified as H&Y stage 2 (74.63%), followed by stage 3 (17.91%), stage 1 (2.99%), stage 4 (2.99%), and stage 5 (1.49%). The mean LED was 861.97 ± 384.34 mg/day (median = 825; range: 113–1866 mg/day).

### 3.2. Demographic and Clinical Characteristics

Demographic and clinical characteristics of PD and non‐PD patients with constipation are summarized in Table 1. Gender distribution was similar between groups (*p* = 0.636). Cognitive impairment was observed in 31.34% of PD patients but was not reported in the non‐PD group, representing a statistically significant difference (*p* < 0.001). Most participants in both groups reported adequate water intake (≥ 1800 mL/day), were nonsmokers, and abstained from alcohol, with no significant differences between groups.

**TABLE 1:** Comparative analysis of demographic and clinical characteristics between PD and non‐PD patients with constipation.

Variable	PD, *n* (%)	Non‐PD, *n* (%)	*p* value
Gender			
• Male	27 (40.30)	18 (36.00)	0.636^2^
• Female	40 (59.70)	32 (64.00)	
Cognitive impairment	21 (31.34)	0	< 0.001^1^
Adequate water intake (≥ 1800 mL/day)	66 (98.51)	50 (100)	1.000^1^
Coffee intake	20 (29.85)	23 (46.00)	0.073^2^
Nonsmoking	67 (100)	49 (98.00)	0.427^1^
Nonalcohol drinking	63 (94.03)	43 (86.00)	0.201^1^
Exercise	47 (70.15)	28 (56.00)	0.114^2^
History of gastrointestinal problems			
• Yes (*n* = 35)	20 (29.85)	15 (30.30)	0.986^2^
• GERD	17 (85.00)	3 (20.00)	< 0.001^1^
• Gastric or duodenal ulcer	6 (30.00)	12 (80.00)	0.006^1^
• Internal hemorrhoid	1 (5.00)	0	1.000^1^
Comorbidities			
• Yes (*n* = 86)	53 (79.10)	33 (66.00)	0.112^2^
• Diabetes mellitus	8 (15.09)	9 (27.27)	0.168^2^
• Hypertension	33 (62.26)	23 (69.70)	0.482^2^
• Hyperlipidemia	41 (77.36)	20 (60.61)	0.096^2^
• Cancer	2 (3.77)	1 (3.03)	1.000^1^
• Cardiovascular diseases	9 (16.98)	7 (21.21)	0.624^2^
• Cerebrovascular disease	12 (22.64)	5 (15.15)	0.396^2^
• Renal diseases	8 (15.09)	3 (9.09)	0.519^1^
• Liver diseases	3 (5.66)	7 (21.21)	0.040^1^
Use of laxatives	56 (83.58)	46 (92.00)	0.264^1^
Use of more than one laxative drug	19 (28.36)	13 (26.00)	0.777^2^
Digital assistance	2 (2.99)	4 (8.00)	0.400^1^

^1^Fisher’s exact test.

^2^Chi‐squared test.

Coffee consumption was lower among PD patients (29.85%) compared to that of non‐PD patients (46.00%), though the difference did not reach statistical significance (*p* = 0.073). A greater proportion of PD patients engaged in regular physical exercise (70.15%) than non‐PD patients (56.00%), but this difference was also not statistically significant (*p* = 0.114).

The prevalence of gastrointestinal comorbidities varied between groups. While the overall proportion of participants with gastrointestinal conditions was similar, specific diagnoses differed. Gastroesophageal reflux disease (GERD) was significantly more prevalent among PD patients (85.00%) compared to that of non‐PD patients (20.00%; *p* < 0.001). In contrast, gastric or duodenal ulcers were more commonly reported by non‐PD patients (80.00%) than among PD patients (30.00%; *p* = 0.006).

Although the overall prevalence of underlying comorbidities did not differ significantly (*p* = 0.1122), liver disease was more frequently reported in the non‐PD group (21.21%) than in the PD group (5.66%; *p* = 0.040). There were no significant between‐group differences in the use of laxatives or the need for digital/manual assistance during defecation.

### 3.3. Dietary Intake and Exercise Habits

Table 2 compares the dietary intake and exercise patterns between PD and non‐PD patients with constipation. The mean daily water intake was slightly higher among PD patients (1800 ± 394.35 mL) than among non‐PD patients (1740 ± 476.41 mL), but the difference was not statistically significant (mean difference = 60 mL; 95% CI: −22224.10–104.10; *p* = 0.470).

**TABLE 2:** Comparison of daily intake of water, coffee, carbohydrate, and fiber, as well as exercise habits, between PD and non‐PD patients with constipation.

Variable	PD	Non‐PD	Diff.	95% CI	*p* value
Water intake (mL per day)					
• Mean (SD)	1800 (394.35)	1740 (476.41)	60	−224.10–104.10	0.470^1^
• Median (Range)	2000 (500–3000)	1750 (1000–2500)			
Coffee intake (cups per month)					
• Mean (SD)	28 (8.80)	27.92 (3.88)			
• Median (Range)	30 (20–60)	30 (20–30)	0	0‐0	0.414^2^
Carbohydrate intake (grams per day)					
• Mean (SD)	57.09 (14.50)	62.08 (10.56)			
• Median (Range)	52 (20–82)	54 (52–90)	2	2–4	0.003^2^
Fiber intake per day (%)					
• Mean (SD)	29.40 (9.79)	26.40 (6.31)			
• Median (Range)	30 (10–70)	30 (10–40)	0	0‐0	0.084^2^
Frequency of exercise (days per week)					
• Mean (SD)	5.55 (1.43)	5.04 (0.96)	0.52	−1.12–0.09	0.093^1^
• Median (Range)	5 (3–7)	5 (3–7)			
Duration of exercise (minutes per day)					
• Mean (SD)	30.53 (14.07)	30.00 (11.78)			
• Median (Range)	30 (10–60)	30 (15–60)	0	0‐0	0.971^2^

*Note:* Diff. = mean difference/median difference.

^1^Independent *t*‐test.

^2^Mann–Whitney test.

Coffee consumption was comparable between the two groups, with a median intake of 30 cups per month, and no significant difference observed (*p* = 0.414). In contrast, carbohydrate intake differed significantly: non‐PD patients consumed a higher amount (62.08 ± 10.56 g/day) compared to PD patients (57.09 ± 14.50 g/day), with a median difference of 2 g (95% CI: 2–4; *p* = 0.003).

Fiber intake was marginally higher among PD patients (29.40 ± 9.79%) compared to that of non‐PD patients (26.40 ± 6.31%), though the difference did not reach statistical significance (*p* = 0.084).

Exercise frequency also tended to be greater in the PD group (mean = 5.55 ± 1.43 days/week) compared to that in the non‐PD group (5.04 ± 0.96 days/week), with a mean difference of 0.52 days (95% CI: −1.12–0.09; *p* = 0.093). The average duration of daily exercise was similar in both groups (approximately 30 min/day), with no significant difference noted (*p* = 0.971).

### 3.4. CSS Outcomes

**FIGURE 1:**
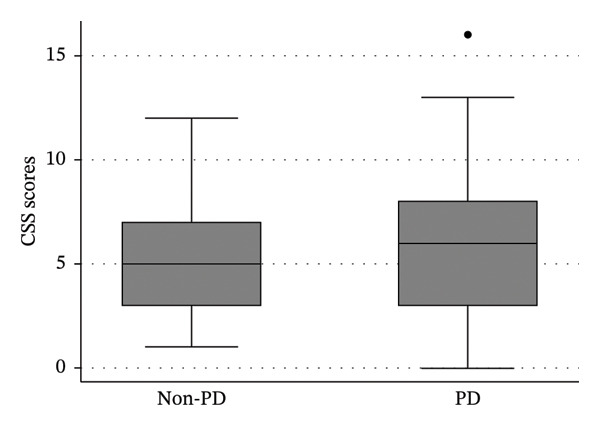
Total Constipation Scoring System (CSS) scores after treatment in patients with and without Parkinson’s disease.

The mean total CSS scores following treatment did not differ significantly between PD and non‐PD patients (PD: 6.07 ± 3.57; median = 6; range: 0–16 vs. non‐PD: 5.24 ± 2.84; median = 5; range: 1–12; *p* = 0.172) (Figure 1). Post‐treatment constipation severity classifications and individual domain scores are summarized in Table 3.

**TABLE 3:** Comparison of constipation severity and symptom domain scores between PD patients and non‐PD patients after constipation treatment.

Variable	PD, *n* (%)	Non‐PD, *n* (%)	*p* value
CSS score (30 scores)			
• Mild constipation (1–10)	58 (86.57)	48 (96.00)	0.113^3^
• Moderate constipation (11–20)	9 (13.43)	2 (4.00)	
• Severe constipation (21–30)	0	0	
Frequency of bowel movements (score 0–1)	65 (97.01)	47 (94.00)	0.650^1^
• 0‐ 1–2 times/1–2 days	35 (52.24)	19 (38.00)	0.282^3^
• 1‐ 2 times/week	30 (44.78)	28 (56.00)	
• 2‐ 1 time/week	2 (2.99)	3 (6.00)	
• 3‐ < 1 time/week	0	0	
• 4‐ < 1 time/month	0	0	
Difficulty score (0–1)	52 (77.61)	38 (76.00)	0.838^2^
• 0‐ No	29 (43.28)	19 (38.00)	0.681^3^
• 1‐ Rarely	23 (34.33)	19 (38.00)	
• 2‐ Occasionally	13 (19.40)	12 (24.00)	
• 3‐ Frequently	2 (2.99)	0	
• 4‐ Always	0	0	
Completeness score (0–1)	55 (82.09)	40 (80.00)	0.814^1^
• 0‐ No	30 (44.78)	19 (38.00)	0.678^3^
• 1‐ Rarely	25 (37.31)	21 (42.00)	
• 2‐ Occasionally	12 (17.91)	9 (18.00)	
• 3‐ Frequently	0	1 (2.00)	
• 4‐ Always	0	0	
Abdominal pain score (0–1)	55 (82.09)	45 (90.00)	0.294^1^
• 0‐ No	30 (44.78)	28 (56.00)	0.398^3^
• 1‐ Rarely	25 (37.31)	17 (34.00)	
• 2‐ Occasionally	12 (17.91)	5 (10.00)	
• 3‐ Frequently	0	0	
• 4‐ Always	0	0	
Time score (0–1)	62 (92.54)	50 (100)	0.070^1^
• ‐ < 5	40 (59.70)	37 (74.00)	0.143^3^
• 1‐ 5–10	22 (32.84)	13 (26.00)	
• 3‐ 11–20	4 (5.97)	0	
• 2‐ 21–30	0	0	
• 4‐ > 30	1 (1.49)	0	
Defecation without assistance (0‐1)	59 (88.06)	45 (90.00)	0.741^2^
• 0‐ No	14 (20.90)	4 (8.00)	0.146^4^
• 1‐ Laxatives	45 (67.16)	41 (82.00)	
• 2‐ Suppo or maneuver	8 (11.94)	5 (10.00)	
Failure score (0–1)	67 (100)	50 (100)	—
• 0‐ No	56 (83.58)	39 (78.00)	0.445^3^
• 1‐ 1–3	11 (16.42)	11 (22.00)	
Duration of constipation score (0–1)	26 (38.81)	43 (86.00)	< 0.001^1^
• 0‐ No	4 (5.97)	11 (22.00)	< 0.001^4^
• 1‐ 1–5	22 (32.84)	32 (64.00)	
• 2‐ 6–10	34 (50.75)	7 (14.00)	
• 3‐ 11–20	7 (10.45)	0	
• 4‐ > 20	0	0	

^1^Independent *t*‐test.

^2^Mann–Whitney test.

^3^Fisher’s exact test.

^4^Chi‐squared test.

According to CSS classification, the majority of patients in both groups were categorized as having mild constipation (CSS score 1–10), with 86.57% in the PD group and 96.00% in the non‐PD group. A greater proportion of PD patients (13.43%) were classified as having moderate constipation (score 11–20) compared to non‐PD patients (4.00%), although this difference was not statistically significant (*p* = 0.113).

No significant differences were observed between groups across multiple CSS symptom domains, including frequency of bowel movements, difficulty in evacuation, sense of incomplete evacuation, abdominal pain, time spent on defecation, and the use of assistance (e.g., laxatives, suppositories, or manual maneuvers), with all *p* values > 0.05.

A notable finding was a significant difference in the duration of constipation symptoms. A higher proportion of non‐PD patients (86.00%) scored positively in this domain, indicating recent‐onset symptoms, whereas only 38.81% of PD patients did so (*p* < 0.001). In contrast, PD patients more frequently reported long‐standing symptoms, with 50.75% indicating a duration of 6–10 years and 10.45% reporting symptoms lasting 11–20 years.

No patients in either group were classified as having severe constipation (CSS score 21–30). All participants scored within the normal range in the domain assessing failure to defecate, suggesting treatment was effective in this aspect.

### 3.5. Laxative Use Patterns

Table 4 compares the patterns of laxative use between PD and non‐PD patients with constipation. Sennosides were the most commonly used agent in both groups, reported by 67.16% of PD patients and 62.00% of non‐PD patients, with no significant difference observed (*p* = 0.562). However, several other agents demonstrated differential usage between groups. Lactulose was used significantly more frequently by non‐PD patients (34.00%) compared to that of PD patients (14.93%; *p* = 0.015). Similarly, mucilin (a bulk‐forming fiber supplement) was more commonly used among non‐PD patients (20.00%) than among PD patients (4.48%; *p* = 0.015). In contrast, milk of magnesia and Unison enema were used exclusively or more frequently by PD patients (7.46% and 10.45%, respectively), with no reported use among non‐PD patients. The use of Unison enema was significantly higher in the PD group (*p* = 0.020), while the difference in milk of magnesia use approached statistical significance (*p* = 0.070). The use of bisacodyl and herbal remedies was infrequent across both groups, with no significant differences noted (*p* = 1.000 and *p* = 0.506, respectively). These findings suggest that although sennosides remain the most frequently used laxative in both groups, preferences for specific osmotic, bulk‐forming, and enema‐based agents may differ according to PD status, potentially reflecting differences in symptom patterns or treatment responses.

**TABLE 4:** Comparison of laxative use in clinical practice between PD and non‐PD patients with constipation.

Laxative drug	PD, *n* (%)		Non‐PD, *n* (%)		*p* value
	Use	Not use	Use	Not use	
Sennosides	45 (67.16)	22 (32.84)	31 (62.0)	19 (38.00)	0.562^1^
Lactulose	10 (14.93)	57 (85.07)	17 (34.00)	33 (66.00)	0.015^1^
Milk of magnesia	5 (7.46)	62 (92.54)	0	50 (100.00)	0.070^2^
Bisacodyl	2 (2.99)	65 (97.01)	1 (2.00)	49 (98.00)	1.000^2^
Mucilin	3 (4.48)	64 (95.52)	10 (20.00)	40 (80.00)	0.015^2^
Unison enema	7 (10.45)	60 (89.55)	0	50 (100.00)	0.020^2^
Herbal medicine	2 (2.99)	65 (97.01)	0	50 (100.00)	0.506^2^

^1^Fisher’s exact test.

^2^Chi‐squared test.

## 4. Discussion

Constipation is a frequent nonmotor symptom in patients with PD and significantly interferes with daily functioning and quality of life. Neurologists and internists often consider it a complex and challenging symptom to manage. Previous studies have reported the high prevalence of constipation in PD [8, 9], its associated factors [10], and various treatment strategies [11]. However, evidence describing post‐treatment outcomes under routine clinical practice—particularly in observational settings—remains limited, representing the primary contribution of the present study rather than the introduction of novel therapeutic approaches.

This study aimed to assess treatment outcomes using the CSS to compare PD and non‐PD patients. Contrary to our initial hypothesis, post‐treatment CSS scores were comparable between groups; however, given the observational design and absence of multivariable adjustment, these findings should be interpreted descriptively rather than as evidence of equivalent treatment, effectiveness, or difficulty.

Baseline characteristics showed no significant differences in mean age or gender distribution between the two groups. Regarding nonpharmacological interventions, both groups had comparable habits in terms of water and coffee consumption, fiber intake, and exercise frequency. Notably, approximately half of the participants in both groups reported drinking more than 1800 mL of water per day. This contrasts with a prior study from Japan, where PD patients consumed less than 1000 mL/day [12], and may reflect the effectiveness of health education efforts at our institution. Furthermore, contrary to a study from Shanghai reporting reduced exercise in PD patients due to bradykinesia [9], our PD cohort maintained regular physical activity. This may be explained by the fact that most patients in our study were in the mild to moderate stages of PD and remained responsive to dopaminergic therapy.

Differences in coexisting medical conditions were also observed. Non‐PD patients were more likely to have other gastrointestinal disorders, while cognitive impairment was more prevalent among PD patients. These differences likely reflect referral patterns within tertiary care settings and introduce potential selection bias, limiting the generalizability of findings. Although GERD is recognized in PD and has been associated with vagal dysregulation [13, 14], our study included too few GERD cases to permit meaningful statistical analysis.

Several instruments are available to assess constipation severity, including the Symptom Severity Score, Patient Assessment of Constipation–Quality of Life (PAC‐QOL), and Patient Assessment of Constipation–Symptoms (PAC‐SYM) [15]. However, validated Thai versions are unavailable. Therefore, the CSS, developed by Agachan et al., was used due to its simplicity and established reliability. A key limitation of this approach is the absence of baseline CSS measurements, which precludes assessment of within‐patient symptom improvement and limits interpretation of post‐treatment comparisons. In this study, most participants in both groups fell within the mild CSS category, suggesting generally adequate symptom control. Although a higher proportion of PD patients were classified in the moderate range, this difference did not reach statistical significance.

Although total post‐treatment CSS scores were similar, PD patients reported a significantly longer duration of constipation. This aligns with the existing literature suggesting that constipation often precedes the onset of motor symptoms in PD. For example, Savica et al. reported an odds ratio of 2.48 for constipation occurring prior to PD diagnosis compared with controls [16]. A nationwide study in China similarly found that individuals with constipation were at a higher risk of developing PD [17]. Other reports have estimated that constipation may precede motor symptoms by over a decade [9, 17, 18]. In our study, the majority of PD patients reported constipation durations between 6 and 10 years, consistent with prior findings from Shanghai, which reported an average prodromal period of 6.62 ± 9.32 years [9].

Analysis of laxative use revealed differing prescribing patterns between neurologists and gastroenterologists. While sennosides were commonly used in both groups, non‐PD patients more frequently received lactulose and mucilin (a bulk‐forming agent), whereas PD patients were more often treated with Unison enemas and milk of magnesia (osmotic agent). This heterogeneity in treatment strategies reflects real‐world clinical practice but further limits causal interpretation of treatment outcomes. Although macrogol and lubiprostone have demonstrated efficacy in randomized controlled trials for treating constipation in PD [19–21], they were not available at our institution during the study period. Nonetheless, our findings suggest that commonly available, general‐use laxatives can still be effective in managing constipation in PD patients.

Several limitations warrant consideration. Functional gastrointestinal testing was not performed, limiting the interpretation of physiological findings. Selection bias may arise from recruitment from specialized clinics. Most importantly, the observational design, lack of baseline CSS assessments, treatment heterogeneity, and absence of multivariable adjustment substantially limit causal inference. Despite these constraints, the findings suggest that constipation can be managed to a comparable symptomatic level in patients with early‐to‐moderate PD under routine clinical care. Future prospective studies incorporating baseline measurements, standardized treatment protocols, and multivariable analyses are needed to better define treatment trajectories and optimize management strategies.

## 5. Conclusion

In this observational study conducted under routine clinical management, post‐treatment total CSS scores were comparable between patients with PD and those without PD. Patients with PD reported a longer duration of constipation symptoms, consistent with evidence that constipation may precede the onset of motor manifestations in PD. However, given the observational design, absence of multivariable adjustment, and lack of baseline CSS assessments, these findings should be interpreted with caution and considered descriptive rather than causal. The results underscore the importance of early recognition and individualized management of constipation in PD. Further prospective studies incorporating baseline measurements, standardized treatment protocols, and multivariable analyses are needed to better clarify treatment outcomes and inform clinical practice.

## Author Contributions

Kulthida Methawasin served as the principal investigator and corresponding author, drafted the manuscript, and performed data analysis. Kitsarawut Khuancharee contributed to the drafting and validation of the methodology and participated in data analysis. Atip Krittayasingh was a coinvestigator and coauthor. Piyanant Chonmaitree was the lead investigator from the gastroenterology department. Monton Wongwandee assisted with data collection from the Parkinson’s disease clinics.

## Funding

This work was funded by the Faculty of Medicine, Srinakharinwirot University, 444/2562.

## Disclosure

All authors reviewed and approved the final manuscript. This manuscript was a poster presentation in the International Congress of Parkinson’s disease and Movement Disorders 2022.

## Ethics Statement

This study was approved by the Ethics Committee of Srinakharinwirot University (SWUEC/E‐410/2561). All participants were informed about the study by nurses from the neurology and gastrointestinal clinics and received written participant information sheets. All participants provided written informed consent before their inclusion in the study.

## Conflicts of Interest

The authors declare no conflicts of interest.

## Supporting Information

STROBE statement.

## Supporting information


**Supporting Information** Additional supporting information can be found online in the Supporting Information section.

## Data Availability

The data that support the findings of this study are openly available in https://doi.org/10.17605/OSF.IO/5QX7F at https://osf.io/kc874.
